# Simultaneous transcutaneous electrical nerve stimulation mitigates simulator sickness symptoms in healthy adults: a crossover study

**DOI:** 10.1186/1472-6882-13-84

**Published:** 2013-04-15

**Authors:** Hsin Chu, Min-Hui Li, Yu-Cheng Huang, Shih-Yu Lee

**Affiliations:** 1 Institute of Aerospace and Undersea Medicine, School of Medicine, National Defense Medical Center, Taipei, Taiwan; 2 Department of Neurology, Tri-Service General Hospital, National Defense Medical Center, Taipei, Taiwan

**Keywords:** Transcutaneous electrical nerve stimulation, Motion sickness, Simulator sickness, Crossover, Autonomic nervous system, Heart rate variability, Salivary biomarker, Alpha amylase, Cortisol

## Abstract

**Background:**

Flight simulators have been used to train pilots to experience and recognize spatial disorientation, a condition in which pilots incorrectly perceive the position, location, and movement of their aircrafts. However, during or after simulator training, simulator sickness (SS) may develop. Spatial disorientation and SS share common symptoms and signs and may involve a similar mechanism of dys-synchronization of neural inputs from the vestibular, visual, and proprioceptive systems. Transcutaneous electrical nerve stimulation (TENS), a maneuver used for pain control, was found to influence autonomic cardiovascular responses and enhance visuospatial abilities, postural control, and cognitive function. The purpose of present study was to investigate the protective effects of TENS on SS.

**Methods:**

Fifteen healthy young men (age: 28.6 ± 0.9 years, height: 172.5 ± 1.4 cm, body weight: 69.3 ± 1.3 kg, body mass index: 23.4 ± 1.8 kg/m^2^) participated in this within-subject crossover study. SS was induced by a flight simulator. TENS treatment involved 30 minutes simultaneous electrical stimulation of the posterior neck and the right Zusanli acupoint. Each subject completed 4 sessions (control, SS, TENS, and TENS + SS) in a randomized order. Outcome indicators included SS symptom severity and cognitive function, evaluated with the Simulator Sickness Questionnaire (SSQ) and d2 test of attention, respectively. Sleepiness was rated using the Visual Analogue Scales for Sleepiness Symptoms (VAS-SS). Autonomic and stress responses were evaluated by heart rate, heart rate variability (HRV) and salivary stress biomarkers (salivary alpha-amylase activity and salivary cortisol concentration).

**Results:**

Simulator exposure increased SS symptoms (SSQ and VAS-SS scores) and decreased the task response speed and concentration. The heart rate, salivary stress biomarker levels, and the sympathetic parameter of HRV increased with simulator exposure, but parasympathetic parameters decreased (p < 0.05). After TENS treatment, SS symptom severity significantly decreased and the subjects were more able to concentrate and made fewer cognitive test errors (p < 0.05).

**Conclusions:**

Sympathetic activity increased and parasympathetic activity decreased after simulator exposure. TENS was effective in reducing SS symptoms and alleviating cognitive impairment.

**Trial registration number:**

Australia and New Zealand Clinical Trials Register: http://ACTRN12612001172897

## Background

Simulators are used increasingly not only in research and training, but for consumer entertainment, industry, and medicinal applications as well. Sophisticated motor vehicle and flight simulators that provide realistic perception of movement are used in driving and aviation training. With strict control of environmental conditions, the use of simulators allows for workload assessment or procedural improvement [1]. Simulators have been used for the evaluation or cognitive rehabilitation of patients recovering from anesthesia and those with post-traumatic stress disorders or traumatic brain injury [2,3]. There are many advantages to simulator-based training besides the positive transfer of training effects. For example, compared to driving or flying in a real environment, a test in simulator is safe, economical, and allows for the assessment of participants in a controlled environment, tailoring situations that are sensitive to people with specific needs, and testing participants under objective and repeatable conditions.

Despite all of the advantages of simulators, the issue of motion sickness (MS) in simulator use remains topical. Simulator-induced MS consists of a wide range of symptoms and signs experienced during or after a simulator session and has been referred to as simulator sickness (SS), simulator aftereffects, or simulator adaptation syndrome [4]. The incidence of SS varies widely across simulators and conditions. For fixed-wing and rotary-wing aircraft simulators, the incidence rates of SS range from 10 − 47% and 26 − 69%, respectively [5]. The symptoms and signs of SS include dizziness, spinning sensation, drowsiness, confusion, motor dyskinesia, visual flashbacks, pallor, cold sweating, restlessness, and excessive salivating, and can progress to nausea and eventually emesis. Less commonly described accompaniments of simulator exposure include postural and eye/hand incoordination and sopite syndrome [6,7]. Like MS, many of the signs of SS have been attributed to increased activation of the sympathetic nervous system [8]. It is well established that severe MS is associated with decreased cognitive function and negatively affects psychomotor performance [9,10]. SS may cause stress for those completing assignments on simulators, interfere with the measurement accuracy or effectiveness of training or therapy, lower participant motivation, or even cause complete abandonment of the task. Some of the learned behaviors such as reducing head movement in a flight simulator with the purpose of avoiding SS might not be appropriate or safe for actual flight. Another possible drawback of SS is the potential for dangerous aftereffects. More than 10% of participants will experience pronounced aftereffects such as ataxia, loss of balance, and flashbacks [4,11]. Spatial orientation may also be affected due to imbalance or disequilibrium. The disruption of balance and coordination resulting from exposure to a simulator may be a safety concern, and such aftereffects could be potentially hazardous for simulator users when exiting the simulator or driving home. Unless remedied in some way, SS will limit simulator-based training.

Current evidence for controlling or alleviating SS in simulators is poorly documented. Although there are some quite effective pharmacological interventions for MS, most of the proven effective drugs have some side effects that may affect training efficiency [12]. For example, scopolamine, one of the most promising drugs for MS prevention, may cause sedation, dry mouth, blurring of vision, and lightheadedness [13]. Oral medications for SS treatment might not be feasible, as drug absorption was shown to be impaired due to marked reduction in gastric motility [14]. Another drawback of oral medications is the anticipation of prophylactic need because most peak drug plasma levels are not reached until several hours after administration. To overcome these problems, alternative medicine remedies that are currently used to treat MS should be considered for SS management. Stimulation of Neiguan (P6) acupoint had been evaluated as non-pharmacologic intervention for MS. Although some studies failed to demonstrate significant positive effects [15-17], however, cyclic manual pressure or electrical stimulation to the Neiguan (P6) acupuncture point suppressed MS symptoms of nausea and vomiting in a rotating optokinetic drum paradigm [18]. Transcutaneous electrical nerve stimulation (TENS), a maneuver that alters cardiovascular autonomic responses [19-21], enhances visuospatial abilities and cognitive function [20], had recently been shown to alleviate motion sickness symptoms provoked with Coriolis stimulation [21]. However, the effects of TENS on SS had not been evaluated. The aim of the present study was to investigate the effects of TENS on flight simulator-induced SS.

## Methods

### Design

**Figure 1:**
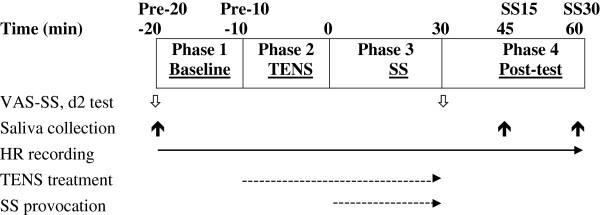
**Schematic illustration of the experimental protocol.** Empty arrows indicate objective assessments and solid arrows indicate subjective assessments. Solid line with arrowhead indicate heart rate recording. Dashed lines with arrowheads indicate transcutaneous electrical nerve stimulation (TENS) treatment or simulator exposure sessions. HR, heart rate; SS, simulator sickness; VAS-SS, Visual Analogue Scales for evaluating Sleepiness Symptoms; Pre-20 and Pre-10, 20 and 10 minutes before simulator exposure, respectively; SS15 and SS30, 15 and 30 minutes after simulator exposure, respectively.

A within-subject crossover design was carried out and the TREND statement was followed [22]. Each subject was asked to complete 4 sessions (control, SS, TENS, and TENS + SS) in a randomized order. A list of random numbers was determined using a computer program (Research Randomizer). Numbers were placed in sealed opaque envelopes before the beginning of the study so that randomization was concealed from the recruiter. Each session consisted of 4 phases: phase 1 (10 minutes), consisting of preparation and recording of baseline physiologic parameters; phase 2 (10 minutes), electrical stimulation (for TENS and TENS + SS sessions); phase 3 (20 minutes), SS stimulation (for SS and TENS + SS sessions) and electrical stimulation (only in TENS + SS sessions); and phase 4 (30 minutes), the post-test period (Figure 1). To compensate for any learned behaviors and to prevent the carryover of intervention, sessions were spaced at least 2 days apart. Data collection and entry was performed by a person blinded to session.

### Participants

Healthy, non-smoking, young male were recruited from trainees taking aviation physiology refresh course in Aviation Physiology Research Laboratory of Gangshan Armed Forces Hospital during July, 2009 and June, 2010 for this study. All subjects were screened for vestibular, visual, cardiovascular, respiratory, and gastrointestinal tract disorders. We calculated sample size using a statistical power analysis program (G*-power) for one tail with a medium effect size, setting statistical significance to α = 0.05 and a power of 0.80. Our calculations yielded an estimate of at least 15 subjects in the group. Each subject provided written informed consent prior to the study. All of the study procedures were in accordance with the declaration of Helsinki and the ethical standards of the human research advisory committee. Subjects were instructed to refrain from the use of medications, alcoholic substances, and caffeinated beverages for 24 hours before and at the time of each study session. They were instructed to be well rested before each study session. All tests were performed between 1:30 PM and 5:00 PM to comply with the schedule of the simulator and to control for possible time-of-day effects.

### Experimental protocol

All test sessions were performed in the DISO trainer in the Aviation Physiology Research Laboratory of Gangshan Armed Forces Hospital. In the Control and TENS sessions, the participants sat quietly in the DISO trainer without activating the simulator while physiological recordings were made. During phase 3 of the SS and TENS + SS sessions, SS was induced with the DISO trainer, a motion-based flight simulator used to simulate a number of illusions and spatial disorientation conditions that are commonly encountered in the aviation environment. The simulator was jointly designed by the Aerospace Physiology Research Laboratory and the Chung-Shan Institute of Science and Technology. The cabin of the spatial disorientation trainer is configurable for the cockpit of F-5E and F-16 fighter aircraft and generic helicopters, with a total of 45 training courses in active and passive mode. It employs a 200 × 200 km terrain, landform, and surface feature board database. Visual display of the simulator is supported with a 3-channel 120° (H) × 30° (V) Collimated Display System for improved field of depth. This device incorporates a 6 degree of freedom (pitch, roll, yaw, heave, surge, and sway) electric pneumatic motion platform (E-Cue 636–4500, Fokker Controls, Netherlands) with a 360° rotational axis (AMST-System technik GmbH, Austria) capable of generating adequate linear and rotatory motion cues to simulate possible flight environments and disorienting visuovestibular conditions. For the current study, 8 flight profiles were chosen: take-off illusion, weather condition, false horizon, light confusion, the leans, autokinesis, semicircular canal defect, and Coriolis illusion. Each subject may request the simulation session be terminated if they found the SS symptoms to be unbearable; otherwise, the entire duration of the simulation (approximately 30 minutes) was completed.

### Intervention

For the TENS and TENS + SS sessions, 30 minutes (complete duration of phases 2 and 3) of TENS was administered at the midline posterior nuchal region (1.5 cm lateral to the seventh cervical vertebra spinous process) and right Zusanli acupoint (located 1 finger breadth lateral to the tibial anterior crest, about 4 finger widths below the knee). The electrical stimulation was conducted in the simulator using a self-contained, battery-operated, dual channel transcutaneous electrical nerve stimulator (SW320, SHINMED, Taiwan, ROC) at a pulse rate of 100Hz. Per the manufacturer’s instructions, the participants set the level of intensity (1 through 10) at the highest comfortable setting, as the intensity setting varies individually. In SS session, placebo-TENS was applied in which the TENS electrodes were in place but no stimulation was given.

### Outcome measures

Prior to test session, MS susceptibility of all subjects was evaluated with the Motion Sickness Susceptibility Questionnaire (MSSQ), the validated Chinese version of the original questionnaire [23,24]. The questionnaire recorded participants’ previous everyday life MS experiences (e.g., different transportation methods and amusement park rides) during childhood (before age 12, subscale MSSQ-A) and adulthood (after age 12, subscale MSSQ-B). Frequencies of exposure to potential MS-provoking vehicles and symptoms were evaluated using a 5-point scale. Outcome indicators including SS symptoms and subjective sleepiness were evaluated with the Simulator Sickness Questionnaire (SSQ) and Visual Analogue Scales for evaluating Sleepiness Symptoms (VAS-SS) before phase 1 and after phase 3 of the experiment, respectively [25]. Cognitive function was evaluated with the d2 test [26].

The SSQ is a well-validated self-report symptom checklist designed to detect the prevalence and severity of 16 possible symptoms associated with SS (general discomfort, fatigue, headache, eyestrain, difficulty focusing, increased salivation, sweating, nausea, difficulty concentrating, fullness of head, blurred vision, dizzy with eyes open, dizzy with eyes closed, vertigo, stomach awareness, and burping) [27]. The degree of severity was rated using a 4-point scale ranging from 0 to 3 (not present, slight, moderate, and severe). The Nausea subscale (general discomfort, increased salivation, sweating, nausea, difficulty concentrating, stomach awareness, and burping), Oculomotor subscale (general discomfort, fatigue, headache, eyestrain, difficulty focusing, difficulty concentrating, and blurred vision), and the Disorientation subscale (difficulty focusing, nausea, fullness of head, vertigo, dizzy with eyes open, dizzy with eyes closed, and blurred vision) provide diagnostic information about particular symptom categories. The overall SS score and the subscale scores are obtained by first summing the values for symptoms in the specific subscale then multiplying by a unique weighting factor. The VAS-SS is used to assess sleepiness. The test requires each subject to assess “how did you feel in the last 10 minutes” with respect to 10 sleepiness symptoms: to have tired eyes, to have heavy eyelids, difficulty to direct one’s eyes, difficulty to maintain open eyes, yawns, motor difficulties, strong sleepiness, to feel dizzy, difficulty to direct one’s attention, brief and involuntary microsleeps. For each symptom, the subjects responded by making a stroke with a pen on a 100-mm line with the left end indicating “not at all” and the right end indicating “very much.” The distance in mm of the mark measured from the left end of the line was used as a dependent variable.

The d2 test [26], a standardized paper-and-pencil test, was utilized to assess cognitive function, more specifically the visual attention before (during phase 1) and after SS induction (after phase 3) in each session. The test form consisted of 14 test lines in a landscape paper layout with 47 characters in each line. Each character consisted of a letter, “d” or “p,” marked with 1, 2, 3, or 4 small dashes. The respondent was required to scan the lines and cross out all occurrences of the letter “d” with 2 dashes while ignoring all other characters. Two types of errors are calculated: errors of omission (missing characters that should have been crossed out) and errors of commission (crossing out characters that should not have been crossed out). The test results included the following norm-referenced scores: the total number of items processed ([TN], the sum of all items processed both correctly or incorrectly), a highly reliable measure of processing speed; percentage of errors ([E%], the proportion of errors made across all processed items), a measurement of the qualitative aspects of performance; the total number of items processed minus errors (TN-E), an indication of the implications of the combined speed and accuracy scores for attention and inhibitory control; and concentration performance ([CP], the number of correctly processed items minus errors of commission), a measure of the distortion in response style. Prior to commencement of the study, all subjects were fully trained on the d2 test to minimize the learning effect.

### Other measurements

Salivary stress biomarker level was evaluated. Three mixed unstimulated saliva samples were collected with Salivette saliva collecting tubes (Sarstedt, Nümbrecht, Germany) at each of the following time points: at phase 1 (pre-20) and 15 min (SS15) and 30 min (SS30) after SS induction. The samples were stored immediately after collection at 0°C and delivered to the laboratory on the following day, where samples were frozen at −80°C until analysis. On the day of the assay, the samples were thawed completely, supernatants were collected, and sAA activity and cortisol concentrations were measured according to the manufacturer’s recommendations (Salimetrics Salivary alpha-amylase Assay Kit and Salimetrics Salivary Cortisol immunoassay kit, Salimetrics, USA).

The Polar system (RS 800 wrist unit and WearLink transmitter, Polar, USA) was used to record the HR of each participant. HRV was determined by imputing the HR data into the Nevrokard LT-HRV software (Nevrokard Kiauta, d.o.o. Slovenia). Power spectrum analysis in the frequency domain was performed by fast Fourier transformation. The low-frequency power component ([LF], 0.04 − 0.15 Hz), high-frequency power component ([HF], 0.15 − 0.40 Hz), ratio of low-to-high frequency spectra power (LF/HF), high-frequency ratio (HF/LF + HF), and low-frequency ratio (LF/LF + HF) were derived.

### Data analysis

Data were presented as mean ± standard deviation (SD). A considerable skewed distribution was observed for raw HRV and salivary biomarkers data. Therefore, these values were analyzed after logarithmic transformation as recommended [28]. Nonparametric statistics were used to analyze these data. For comparison between sessions, a repeated-measures analysis of variance (ANOVA) was used. Correlations between the SSQ and MSSR were assessed by Spearman rho correlation coefficient calculations. Data analysis was performed using the Statistical Package for the Social Sciences (SPSS; version 19.0, SPSS, USA). Results were considered significant when p < 0.05.

### Ethical approval

Ethical approval was given by the Institutional Review Board in Aviation Medicine Research of Gangshan Armed Forces Hospital on June 25, 2009.

## Results

### Participant enrolment

For the current study, 20 participants were recruited and screened for eligibility and 18 were found to be eligible and enrolled in the study. All 18 subjects were assigned to each study condition. 3 subjects were not able to finish all sessions due to conflict between working and experimental schedule. A total of 15 participants (age: 28.6 ± 0.9 years, height: 172.5 ± 1.4 cm, body weight: 69.3 ± 1.3 kg, body mass index: 23.4 ± 1.8 kg/m^2^) met the inclusion criteria and complied with research protocol during the study period.

### Compliance with trial method

Fifteen healthy young men completed 4 test sessions (control, SS, TENS, TENS + SS) in a randomized order. The adverse events of the intervention were collected at points of contact between study staff and participants. There were no reports of adverse events. All participants completed post-intervention measurements, and all participants' data were included in the intention-to-treat analysis.

**Figure 2:**
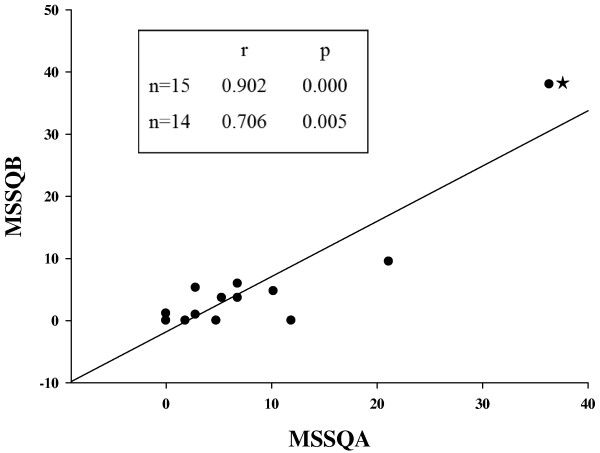
**Correlation between childhood (motion sickness symptom questionnaire [MSSQ]-A) and adulthood (MSSQ-B) scores of motion sickness susceptibility.** ★: high leverage and high influential point [i.e., the “outlier” (36.34, 38.02)]. Inset: correlation coefficients between MSSQ-A and MSSQ-B with (n = 15) and without (n = 14) the outlier.

**Figure 3:**
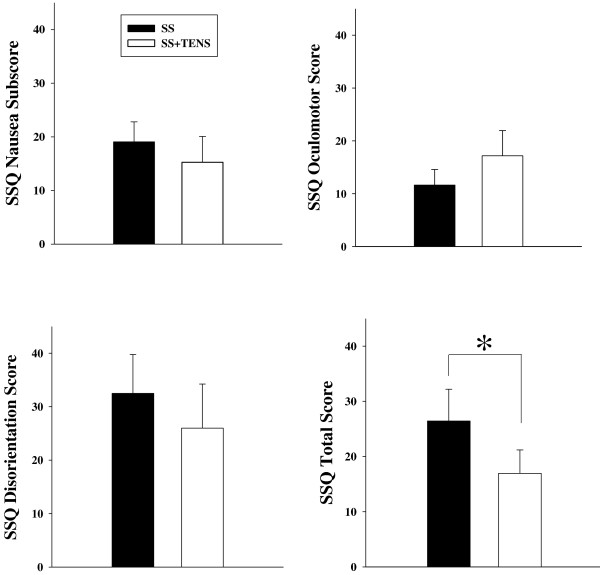
**Simulator sickness symptom rating (SSQ) scores in SS sessions (filled circle) and TENS + SS (empty circle) sessions.** Data were presented as mean ± standard deviation (SD). *p < 0.05.

The mean score of childhood MS susceptibility of the participants (MSSQ-A, 7.49 ± 2.52) was significantly different from (p = 0.032) and correlated with their adult score (MSSQ-B, 4.85 ± 2.48) (r = 0.902, p = 0.000, Figure 2). Regardless of severity, 80% of the participants reported at least 1 SS symptom after simulator exposure. Of all symptoms, increased salivation (n = 12) was most frequently experienced, followed by vertigo (n = 8), fullness of head, eye strain, and dizzy with eyes closed (n = 7). Five participants suffered nausea. The least reported discomforts were sweating (n = 1), stomach awareness (n = 2), and headache (n = 2). In terms of the severity of SS, the most reported symptom severities were “slight,” with only 2 symptoms (eye strain and increased salivation) reported in the “moderate” range. Regarding the profile of the SSQ subscales, disorientation symptoms were predominant, followed by nausea symptoms. The severity of SS as reflected by the total SSQ score increased after the simulator session, which was significantly decreased following TENS intervention (p < 0.05) (Figure 3). Neither childhood nor adult motion sickness susceptibility predict the degree of SS symptom severity (Table 1).

**Table 1:** Intercorrelations between motion sickness susceptibilities and simulator sickness symptom ratings

**Simulator sickness questionnaire (SSQ) scores**				
	**Nausea**	**Oculomotor**	**Disorientation**	**Total**
MSSQ-A	0.059(0.861)	0.120(0.670)	0.240(0.390)	0.242(0.384)
MSSQ-B	−0.039(0.890)	0.133(0.636)	0.227(0.417)	0.228(0.415)

MSSQ-A: Motion Sickness Susceptibility score A for children (<12 years); MSSQ-B: Motion Sickness Susceptibility score B for adults (>12 years). Data are Spearman rho’s correlation coefficients (p-value in parentheses).

An increase of HR was observed after simulator exposure (73.1 ± 4.0 to 78.6 ± 4.2 bpm, p < 0.05, Table 2). HRV analysis revealed that with the development of SS, there was an increase in HR fluctuation, which was reflected in the increase of LF power and a decrease of HF power. The LF/HF ratio also increased with SS (p < 0.05). All of these effects of SS on HRV were eliminated with TENS treatment (Table 2).

**Table 2:** Effects of simulator sickness (SS) and transcutaneous electrical nerve stimulation (TENS) on various physiological parameters

	**Phase 1**		**Phase 2**		**Early post-test**		**Late post-test**	
	**(Pre-20 to pre-10 min)**		**(Pre-10 min to 0 min)**		**(30 min to SS15 min)**		**(SS15min to SS30 min)**	
	**SS**	**SS + TENS**	**SS**	**SS + TENS**	**SS**	**SS + TENS**	**SS**	**SS + TENS**
HR (bpm)	73.09 ± 13.27	76.65 ± 8.95	74.22 ± 14.36	74.22 ± 9.15	77.15 ± 18.10	75.41 ± 9.64*	78.59 ± 13.85^**#**^	76.23 ± 7.10*
LF/(LF + HF)	0.52 ± 0.17	0.60 ± 0.06	0.51 ± 0.18	0.57 ± 0.08	0.70 ± 0.11^**#**^	0.55 ± 0.11*	0.75 ± 0.06^**#**^	0.58 ± 0.10*
HF/(LF + HF)	0.48 ± 0.17	0.40 ± 0.06	0.49 ± 0.18	0.43 ± 0.08	0.30 ± 0.11^**#**^	0.45 ± 0.11*	0.25 ± 0.06^**#**^	0.42 ± 0.10*
LF/HF	1.43 ± 0.87	1.66 ± 0.44	1.37 ± 0.75	1.51 ± 0.50	3.00 ± 1.46^**#**^	1.42 ± 0.57*	3.66 ± 1.74^**#**^	1.68 ± 0.78*

SS: simulation sickness; HR: heart rate; LF: low frequency, HF: high frequency; % change compared with pre-20 min presented in parentheses.

*p < 0.05 relative to SS; ^#^ < 0.05 relative to baseline (Pre-20 min).

Two salivary stress biomarkers, sAA and cortisol, were evaluated to reflect the acute stress responses of the adrenomedullary and adrenocortical systems due to SS, respectively. The sAA activity, but not salivary cortisol concentration, elevated with simulator challenge. TENS did not affect salivary biomarker levels (data not shown).

With the development of SS, the subjects’ self-rating of sleepiness increased and their performance in the d2 test deteriorated, as reflected by elevated VAS-SS scores, smaller TN, increased E%, and poor CP. After TENS treatment, their degree of sleepiness was comparable to baseline levels and the subjects showed increased concentration in the cognitive test with fewer errors. Specifically, processing speed and concentration increased while errors decreased (p < 0.05, Table 3).

**Table 3:** Effects of TENS on attention and sleepiness

	**Control**	**SS**	**TENS**	**TENS + SS**
TN	254.8 ± 41.7	235.6 ± 37.3*	251.6 ± 38.6	257.1 ± 38.9^#^
E%	20.8 ± 22.1	29.9 ± 19.3	22.3 ± 20.2	19.3 ± 19.9^#^
TN-E	210.3 ± 83.6	171.8 ± 74.5*	202.6 ± 77.5	214.6 ± 77.8^#^
CP	254.7 ± 41.7	235.4 ± 37.2*	250.8 ± 38.8	256.8 ± 38.9^#^
VAS-SS	3.2 ± 3.8	10.4 ± 10.1*	4.0 ± 6.0	5.1 ± 5.9^#^

SS, simulator sickness; TENS, transcutaneous electric nerve stimulation.

TN, E%, TN-E, CP are all parameters of the d2 test. TN, Total Number of item processed; E%, Percentage of Errors; TN-E, Total number of items minus error scores; CP, Concentration Performance. VAS-SS, Visual Analogue Scales for evaluating Sleepiness Symptoms.

*p < 0.05 relative to control; ^#^p < 0.05 relative to SS.

## Discussion

In simulators, moving visual scenes and vehicular motion challenge perception, which predisposes individuals to SS. Some SS symptoms such as fatigue, drowsiness, difficulty focusing, and difficulty concentrating affect spatial orientation and cognitive performance [7]. The finding that performance declined in our subjects suffering from SS, in accordance with previous studies in MS models [10,29], suggests that there is a clear need for effective countermeasures [9]. Due to the side effects of pharmacological agents, alternative therapeutic modalities such as acupuncture, acupressure, and electroacupuncture had been tested. Conflicting results have been reported in previous studies in which acupressure or electroacupuncture were applied for the prevention or treatment of MS, with success in some trials [30-32], but not in others [15,16]. The primary finding from this study demonstrated that TENS was effective in reducing SS symptoms and negating the impact of SS on cognitive performance.

The mechanism of TENS is unclear. We simultaneously stimulated 2 sites in our TENS protocol with presumably different mechanism of actions. Neck proprioceptive inputs play a major role in body segment position and orientation in space and during locomotion [33]. Electrical stimulation to the midline posterior nuchal region has been shown to improve visuospatial function in patients [34,35]. The preventive effects of TENS might involve modifying proprioceptive signaling processes by vibratory stimulation of the neck muscles. Electrical stimulation to nuchal spinal segment had direct effects on central nervous system. For example, electrical stimulation of C2 increases activation of the dorsal cochlear nucleus through the somatosensory pathway [36]. Whether other central mechanisms play a role awaits further study. The Zusanli acupoint was chosen as the second acustimulation spot, as acustimulation of this site had been shown to enhance the regularity of gastric myoelectrical activity [37] and was reported to be beneficial in reducing chemotherapy-induced acute vomiting [38]. The arrangement of the TENS protocol in the present study also involved practical considerations. Due to the requirement that the subjects used their hands to fly in the simulator as well as previous reports of inconsistent effects, the Neiguan (P6) acupuncture point was not used as an electrostimulation site.

Past occurrence and severity of MS has been used to predict performance and susceptibility of MS, and a prior history of MS is positively correlated with SS development [39]. In this study, the MSSQ, an instrument validated for the Chinese population, was adopted to evaluate SS susceptibility [23]. Consistent with the published data, the childhood scores (MSSQ-A) were higher than and significantly correlated with the adulthood scores (MSSQ-B), reflecting evolutionary habituation [23,40]. When the high leverage and high influential point (labeled by the star sign in Figure 2) is removed from the analysis, the correlation between MSSQ-A and MSSQ-B become fair but still significant (n = 14; r = 0.706, p = 0.005) (inset of Figure 2). The existence of the outlier was possibly due to: (1) the significant inter-individual variation of motion sickness susceptibility and (2) small sample size. Childhood MS susceptibility (MSSQ-A score) was better correlated with SS symptom severity (SSQ scores; 0.2 < r < 0.6) than adulthood MS susceptibility (MSSQ-B scores; -0.1 < r < 0.1), although neither of these correlations reached statistical significance (data not shown). This finding could be due to small sample size, but may also reflect model-specific sensitivity to MS stimulation, as previously reported [40]. The magnitude of MS susceptibility of our subjects (MSSQ-A, 7.49 ± 2.52; MSSQ-B, 4.85 ± 2.48) was not comparable to published normative data by Golding (28.8 ± 23.3 and 16.7 ± 17 for the MSSQ-A and MSSQ-B, respectively) and Klosterhalfen et al. (both MSSQ-A and MSSQ-B scores, ~20) [23,24]. Possible explanations for these differences include differences in subject numbers, ethnicity, gender, test modality, and experimental designs. Our subjects were all males, while Golding and Klosterhalfen et al. included female subjects (M:F, 70:77 and 37:45, respectively). The MSSQ scores of the current study were lower, which is likely because women are more susceptible to MS than men and/or that men had the tendency to underreport their MS experiences [23,24].

The severity of SS in the current study can be evaluated by total SSQ scores. Scores greater than 20 indicate sufficient discomfort [41]. Another method to quantify SS severity is by comparing current data with values from calibration samples where original SSQ was derived [27]. Total SSQ score of 15 would represent 75th percentile point in a database of more than 1,100 SSQs from healthy subjects pooled from ten flight simulators. The total SSQ score (26.4 ± 5.7) in the current study would correspond to about 88th percentile points in the pooled samples. The scores of the disorientation subscale (32.5 ± 7.2; >90th percentile) were the highest among all of the subscales, followed by nausea subscores (19.1 ± 3.7; about 85th percentile) and oculomotor subscores (11.6 ± 2.9; 69th percentile). Disorientation in the simulator is likely caused by the pseudo-Coriolis stimuli engendered from side-to-side head movements in the simulator and self-motion produced by visual cues [42]. Disorientation, loss of balance, and ataxia are common problems noted by trainees and subjects after exiting a dynamic simulator. High disorientation scores are correlated with postural instability, which may be a safety concern for subjects after exiting the simulator who may need to walk, climb stairs, drive, or even fly an airplane [43].

A quantitative relationship between subjective SS and objective physiological measurements of the central and autonomic nervous systems have been reported [44]. For example, HR has been reported to change from baseline levels following simulator exposure [45]. HR can be modified by parasympathetic fibers and sympathetic fibers. The effects of sympathetic and parasympathetic activity on HR can be monitored with the LF/LF + HF and HF/LF + HF ratios of HRV, respectively. The LF/HF ratio is used for estimating the overall balance between the sympathetic and parasympathetic nervous systems. A higher value indicates increased sympathetic activity or reduced parasympathetic activity. Previous reports revealed conflicting observations about the HR responses to motion sickness. HR increases [46,47], decreases [48] or not changed with MS [49]. Possible explanations for the discrepancy include individual variation and susceptibility [47], physical characteristics of the stimuli (different MS models; intensity, frequency and duration of motion) [50]. HR and sympathetic activity (low-frequency ratio) were elevated while parasympathetic activity (high-frequency ratio) was suppressed in current study, with resultant dominance of sympathetic over parasympathetic activity (increased LF/HF ratio). These effects of SS were all eliminated with TENS treatment (Table 2).

Simultaneous TENS treatment significantly ameliorated SS symptoms, as reflected by the decrease in the total SS scores. TENS treatment per se did not significantly change HR and HRV parameters (data not shown). One possible mechanism by which TENS affects SS is by counteracting certain pathways that are stimulated by simulator exposure. The finding that TENS effectively ameliorated SS symptoms in parallel with its effect on HRV supports this hypothesis. Some other studies failed to demonstrate treatment effects of electrical or acupoint stimulation for MS symptoms. Possible explanations for such disparity include placebo effects, stimulation sites, experimental models, participants characteristics (for example, gender, ethnicity).

MS is associated with increased excretion of cortisol. Epinephrine and noradrenaline are also elevated and may reflect a nonspecific stress response [51]. Basal levels of salivary stress biomarkers were correlated with MS tolerance or susceptibility in previous experimental paradigms [52,53]. However, in our subjects, basal cortisol concentrations and sAA activity levels were not associated with the severity of SS symptoms (i.e., SSQ scores), and hence were not associated with SS tolerance (data not shown). One possible cause of this discrepancy is gender, as cortisol had been shown to predict MS tolerance in women but not in men [54]. Another potential confounder is the model used to induce MS. A previous study that used basal sAA levels to predict MS susceptibility was conducted in parabolic flight, a markedly stronger stimulus than a flight simulator. The sAA activity increased after the simulator sessions, although the levels did not reach statistical significance. Gordon et al. reported that sAA activity was significantly higher in subjects that were susceptible to seasickness relative to non-susceptible subjects [55]. Our results could be explained by low susceptibility (i.e., lower MSSQ scores) of our subjects, as stated above. Differences in experimental models and design could also explain these differences.

The present study has some limitations that should be acknowledged. The flight simulator used in current study is used to train military personnel. The training schedule of the simulator is very tight. Thus, including a larger sample size is not feasible. The small sample size and inclusion of only young male subjects preclude our ability to generalize our results to average SS sufferers. Physiological mechanisms of SS and responses to different simulators might not be universal. For example, SS induced by motion-based simulators and Coriolis stimulation are categorized as type 1 neural mismatch due to visual-vestibular conflict, but SS associated with fixed-based simulators and “Cinerama/Imax sickness" are categorized as type 2a neural mismatch [50]. Closing the eyes stops the perceived motion and dramatically reduces vection-induced SS in fixed-based flight simulators but not motion-based simulators [27]. Therefore, whether TENS treatment can be applied to SS induced by other simulators such as virtual reality awaits future study. In the current study, we did not screen for susceptible subjects in advance, but carried out crossover sessions in randomized orders to decrease the impact of learning or familiarization effects. It is still possible that the effect of TENS could be cancelled out, despite the fact that no significant correlation was found between the MSSQ and SSQ scores. Testing the effect of TENS on simulator-specific SS-susceptible subjects could possibly provide an answer to this question. Approximately 10% of the subjects will experience pronounced aftereffects, including illusory sensations of climbing and turning, perceived inversions of the visual field, and disturbed motor control [11,56]. Although the aftereffects of simulator exposure usually dissipate in 1–2 hours, persistence of these effects lasting more than 6 hours have been reported [11]. Measurements of physiological parameters (HR, HRV, and salivary biomarkers) were performed for only 30 minutes after simulator exposure in current study. The possibility of the existence of extended SS aftereffects cannot be completely ruled out. Thus, we cannot make any claims regarding the effects of TENS on aftereffects. Future studies should strive to monitor SS and the effects of TENS well after the simulator session. Data were collected after completion of all 8 spatial disorientation provocation protocols. Future studies should focus on individual programs to determine if SS is more prevalent or severe following particular maneuvers and the effects of TENS on individual maneuvers.

The results from this study indicate that the incidence and severity of SS after exposure to a simulator are not negligible. These ill effects are characterized by symptoms related to the autonomic nervous system. Simulators are used in occupational training, evaluation, and research. They also allow occupational therapists to conduct assessments or remedial sessions in a controlled environment. Researchers and therapists, however, need to be aware of and able to reduce SS symptoms. We have identified a countermeasure to prevent the ill effects of SS. The likely mechanism of action of this alternative therapy for SS involves modification of autonomic activities. Our findings have clinical and research implications. Future work includes the adjustment of TENS parameters such as the duration and location of electrical stimulation to maximize its effects and to test the efficacy of TENS in other MS/SS models. Controlled trials comparing TENS with other treatment modalities should provide further insight into the optimal method for controlling SS. Such studies will reveal the beneficial effects of TENS on simulator performance and aid researchers and occupational therapists in implementing best practices for their clients receiving simulator-based training or rehabilitation services. Moreover, conducting such studies will conclusively address the causes of SS; only then can effective SS mitigation strategies be developed and tested.

## Conclusions

In summary, cognitive function and autonomic nervous activity were adversely affected by simulator exposure. Preventive TENS was effective in reducing SS symptoms and alleviating cognitive impairment. The beneficial effects of TENS should aid researchers and occupational therapists in implementing best practices for their clients receiving simulator-based training or rehabilitation services.

## Abbreviations

CP: Concentration performance; E%: Percentage of errors; HR: Heart rate; HRV: Heart rate variability; HF: High-frequency power component; HF/LF + HF: Ratio of high-frequency power component; LF: Low-frequency power component; LF/HF: Ratio of low-to-high frequency spectra power; LF/LF + HF: Ratio of low-frequency power component; MS: Motion sickness; MSSQ: Motion Sickness Susceptibility Questionnaire; MSSQ-A: Motion Sickness Susceptibility score A for children (<12 years); MSSQ-B: Motion Sickness Susceptibility score B for adults (>12 years); sAA: Salivary alpha amylase; SS: Simulator sickness; SSQ: Simulator Sickness Questionnaire; SD: Standard deviation; TENS: Transcutaneous electrical nerve stimulation; TN: The total number of items processed; TN-E: The total number of items processed minus error scores; VAS-SS: Visual Analogue Scales for Sleepiness Symptoms.

## Competing interests

The authors declare that they have no competing interests.

## Authors’ contributions

HC conceived of the study, participated in its design and coordination and drafted the manuscript. ML conceived of the study, participated in the design and coordination of the study. YH carried out the experiment and performed the statistical analysis. SL participated in the coordination and execution of the experiments. All authors read and approved the final manuscript.

## Pre-publication history

The pre-publication history for this paper can be accessed here:

http://www.biomedcentral.com/1472-6882/13/84/prepub
